# Mechanism(s) of prolonged attenuation of allergic responses after modulation of idiotypic regulatory network

**DOI:** 10.1186/s13223-019-0393-7

**Published:** 2019-12-04

**Authors:** R. M. Gorczynski, T. Maqbool, G. Hoffmann

**Affiliations:** 1 Universityof Toronto, Toronto, ON Canada; 2 Network Immunology, Vancouver, BC Canada; 3 Cedarlane Labs, Burlington, ON Canada; 4 Toronto, Canada

**Keywords:** Immune network theory, Tregs, Allergic immunity, IL-4, IL-31, Allergic desensitization

## Abstract

**Background:**

We showed previously that allergic reactivity to ovalbumin (OVA) could be regulated in mice following perturbation of immune networks using combinations of an immune Ig along with anti-idiotypic Ig. We have explored features of this regulation including: its persistence after cessation of administration of combined Igs; the ability of heterologous Igs to produce immunoregulation; a role for Treg induction in regulation; and the ability to attenuate responses in mice pre-sensitized to an allergic stimulus.

**Methods:**

BALB/c mice were sensitized to OVA. Mice also received 5 weekly injections of immune Ig or anti-idiotype Ig (at separate sites) from either homologous (mouse) or heterologous (human) sources. In the latter case pooled IVIG (given IM, hence hereafter IMIG) was used as a source of anti-idiotype Ig, and human anti-Tet as immune Ig. Injections of the Ig were given from the time of OVA sensitization (to attenuate development of immunity), or after pre-sensitization of mice (to attenuate existing allergic responses). All mice were assayed for development of OVA-specific serum IgE and IgG, as well as the production of OVA-induced IL-2, IL-4, IL-13, IL-31 and IL-33 in splenocytes cultured for 72 h. In studies examining possible mechanism(s) responsible for inhibition of immunity mice received, in addition to the Ig treatments described, infusion of depleting anti-CD4, and/or anti-CD8 antibodies, or a mAb to TNFSFR25, known to expand Tregs implicated in regulation of Allo immunity.

**Results:**

Combinations of both heterologous and homologous immune Igs and anti-idiotype Igs attenuated OVA allergic responses in both naïve and pre-sensitized mice. This attenuation persisted in mice greater than 14 weeks after cessation of treatment with the Igs used. Finally, depletion of either CD4 or CD8 cells ameliorated the suppressive effect seen, while the combination of anti-CD4 and anti-CD8 essentially abolished suppression. Suppression was further enhanced by anti-TNFSFR25 mAb.

**Conclusions:**

We conclude that the combine Ig treatment protocols used produced a long-lasting suppression of allergic immunity, even in pre-sensitized animals. The effects seem to depend upon induction and expansion of Tregs and represents a novel approach to treatment of allergic disease in humans and other animals.

## Introduction

Allergic diseases constitute a major clinical burden globally [1–3]. Atopic disorders are driven by development of allergen specific immunity through priming of Th2 type cells (releasing IL-4, IL-5 and IL-13) which in turn heighten IgE production by B cells Eosinophil activation and recruitment and bronchial hyperreactivity, are at the forefront of the pathophysiology of disease [4]. More recently other groups have focused also on a role for TH17 cells [5] and their cytokine production, and Tregs [6, 7] as having an important role to play in the evolution of allergic responses in sensitized individuals [8].

Specific subcutaneous immunotherapy (SCIT: often referred to as desensitization therapy) for respiratory allergy and severe allergic reactions has been used for > 70 years [9–11], with good documentation for its efficacy and safety [7, 12] using extracts which have been standardized either biologically or immunologically. Sublingual immunotherapy (SLIT) is a newer in vogue alternative to SCIT, which is also used quite broadly. The mechanisms of action of allergen specific immunotherapy are not well understood though it is evident that early events include mast and basophil cell desensitization, followed by Treg development, including IL-10 secreting Treg [13], altering allergen induced cytokine balance with reduction of Th2 type cytokines [4, 14] and ultimately modulation of B cell IgE production [8].

A major issue in current immunotherapy approaches (SCIT and/or SLIT) involves characterization of the allergen itself. The advent of recombinant molecular approaches has increased hope that hypoallergenic derivatives of the offending agent may become available [15]. Indeed, it may even prove possible to target and expand allergen specific Treg themselves [16]. Interestingly, there is evidence that the sublingual route of allergen delivery has less risk of severe adverse effects anyway [17], likely a result of functional difference between oral antigen-presenting cells (APCs) and Langerhans cells, their skin counterpart. Importantly, it is still unclear whether SLIT (or SCIT) must be given seasonally and or continuously for efficacy to be maintained [18].

Given this background it is evident that there remains an unmet need to develop novel therapies. We have recently reported on the use of a combined injection of polyclonal anti-idiotype antibodies, along with polyclonal immune antibodies, as a treatment aimed at “re-setting” immune regulatory networks in rodents [19]. This treatment led to a marked attenuation of a variety of immune reactivities, including decreased inflammatory cytokines and inflammatory colitis; suppression of skin graft rejection in a transplant model; and decreased IgE and IL-4 production as a marker of attenuation of allergen-induced Th2 activity in a rodent model (sensitization of mice to ovalbumin, OVA). Importantly, the antigen-specific regulation seen was independent of the specific antigens used to prepare the polyclonal immune Ig. One possible mechanism to help explain these effects involved the perturbation of regulatory T cell networks [19–21]. Such a concept is consistent with other data favouring a role for Tregs in control of allergic reactivity [22]. More recently we have extended these studies to show that attenuation of allergic responses (skin reactivity to peanut butter sensitization) can also be suppressed in Beagle dogs using the same methodological approach [23, 24]. Our hypothesis predicted that similar immunoregulation would be seen using treatment not just with immune Ig and anti-idiotype Ig from the same species (homologous), but across species (heterologous Ig treatment) [19–21, 23].

All our data to date has looked at the ability of anti-idiotype Ig and immune Ig to attenuate immunity in a preventative fashion, in BALB/c mice newly exposed to ovalbumin (OVA) as Th2-inducing allergen, or dogs exposed to peanut butter. We have now addressed several novel issues. Firstly, we show a similar attenuation of Th2 immunity to OVA occurs in mice receiving heterologous (human derived) immune Ig and anti-idiotype Ig (see also [23]). In addition, we have documented that even after cessation of Ig treatment, the attenuation of Th2-induced immunity persists > 14 weeks. Finally, in this first series of studies we have shown that desensitization in already sensitized (OVA-immune) mice was achieved using the same protocol.

We have also explored in preliminary fashion the mechanism(s) responsible for the attenuation of Th2 responses seen. Our data show that depletion of both CD4^+^ and CD8^+^ T cells post administration of anti-idiotype and immune Ig abolishes the suppression seen-the effect was most marked with combined CD4 and CD8 depletion. Most interestingly, using mAbs shown elsewhere to expand CD25^+^Treg [22, 25, 26], we document increased suppression of Th2 responses by infusion of these mAbs after first commencing treatment with anti-idiotype and immune Ig. We suggest these data reflect an important role for perturbation of immune regulatory networks using homologous or heterologous anti-idiotype Ig and immune Ig in suppression of Th2 immunity, and thus potentially allergic responses, in naïve and sensitized hosts.

## Materials and methods

### Mice

All mouse experiments were conducted after ethics approval of a local animal care committee, in compliance with the Canadian Council on Animal Care (CCAC). Female BALB/c, C3H and C57BL/6 mice were purchased from Jax Labs, Bar Harbor Maine. All mice were housed 5 per cage and maintained at the Cedarlane animal facility. Mice were used at 8 weeks of age.

### Induction of IgE after ovalbumin (OVA) immunization

**Fig. 1:**
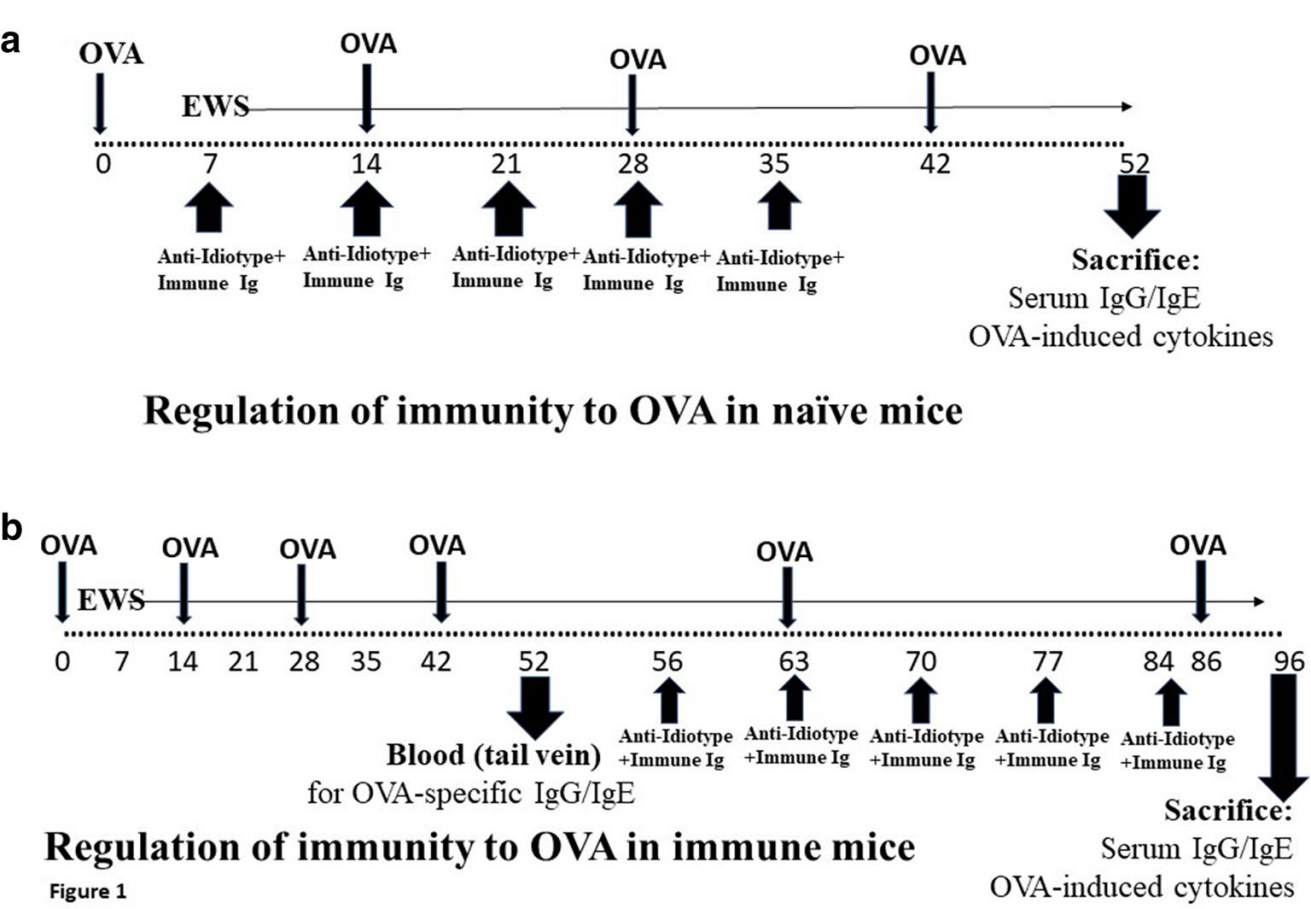
**a** Schematic to show sequence of immunization with OVA in alum (given ip) and anti-idiotype Ig and immune Ig (both given im at separate sites) in naïve mice. All mice received egg white solution (EWS) in their drinking supply from the time shown (day 7). Animals were sacrificed for measure of OVA-specific IgG and IgE and OVA-induced cytokines in 72 h cultures of splenocytes. **b** Similar schematic to show sequence of immunization with OVA in alum (given ip) and anti-idiotype Ig and immune Ig (both given im at separate sites) in mice immunized with OVA before treatment with Igs. All mice received egg white solution (EWS) in their drinking supply throughout study from the time shown (day 7). Tail bleeds were used at 52 days to assess induction of OVA-IgG and IgE, and animals were sacrificed for measure of OVA-specific IgG and IgE and OVA-induced cytokines in 72 h cultures of splenocytes 10 days after a final OVA boost (d 96)

The protocol used to immunize naïve BALB/c mice to produce IgE against OVA was a modification of that described elsewhere [19]. 8 mice per group received 10 μg OVA emulsified in alum intraperitoneally (ip) in 0.2 ml PBS at days 0, day 14, 28 and 42 (see Fig. 1a). Beginning at day 7 of treatment, unless otherwise shown, all mice received egg white solution (EWS) in their drinking water. A control group received no OVA immunization. In some cases, as documented in the text, groups of animals received weekly intramuscular (im) injections, beginning at day 7 post first OVA immunization, of 75 μg of pooled polyclonal anti-idiotype Ig [C3H anti–anti-C3H Ig (C3H anti-BL/6 absorbed with BL/6)] and 10 μg pooled polyclonal BL/6 anti-C3H immune Ig [19]. Other groups received 75 μg of human IMIG or 10 μg human anti-Tet Ig (both from GRIFOLS, Canada [23]). Mice received a further boost of OVA in alum 7 days following treatment with Ig injections, and were sacrificed 10 days after the final immunization (day 52) for analysis of serum OVA-specific IgE/IgG and production of OVA-induced IL-2/IL-4/IL-31/IL-33 over 72 h in culture from stimulated splenocytes (Fig. 1a).

When OVA-sensitized mice were used as subjects for exploration of the effect of anti-idiotype Ig and immune Ig on responses to OVA, the founder mice were initially pre-immunized with OVA (4 injections) and given EWS over a period of 6 weeks (Fig. 1b). 10 days after the last OVA immunization (day 52) samples of blood were harvested from the tail vein (75 μl) of each mouse to assay for OVA-specific IgE and IgG by ELISA (below). Treatment with the combined Igs (again 5× injections) was begun on all mice 56 days following initial OVA immunization (14 days after the last OVA immunization). These mice received two further boosts with OVA in alum at day 63 and day 86 (2 days after the last immunoglobulin treatment) post initiation of study, with sacrifice at day 96 (Fig. 1b).

### Measurement of serum IgE and serum IgG to OVA, and of antigen (or mitogen)-induced cytokine production by splenocytes in ELISA

Blood was obtained from mice by tail vein (day 52 post immunization in studies using pre-sensitized mice) and at sacrifice (days 52/96 using naïve or pre-sensitized mice) by cardiac puncture. In addition, at sacrifice, single cell splenocyte preparations from individual animals were resuspended in alpha MEM with 10% fetal calf serum (αF10) after washing (800*g*×5 min at 4 °C).

OVA-specific IgE or IgG was measured in all serum samples by ELISA using plates coated with 100 ng/well of OVA and developed with HRP-anti-mouse IgE or HRP anti-IgG (ThermoFisher), followed by appropriate substrate.

Sera from mice receiving Human IgG was also tested for the presence of anti-Human IgG responses in ELISA plates coated with human IgG (100 ng/ml: Abcam, San Francisco, USA) and with goat anti-mouse IgG Fab secondary antibody (1:1000 dilution) (ThermoFisher, Canada: Cat 62-6520).

After culture for 72 h of 5 × 10^6^ splenocytes from individual animals in duplicate in vitro in 2 ml αF10 with 0.1 μg/ml OVA, culture supernatants were assayed by commercial ELISA kits for IL-2, IL-4, IL-13, IL-31 and IL-33 (BioLegend, CA, USA) production.

### Statistics

All data from experiments reported below are summed over at least two studies, with a minimum of 16 mice in all groups for all experiments. In general, for studies with multiple groups, a multivariate analysis of variance (MANOVA) test was first applied to assess for any significant differences between groups, and subsequently, where indicated, paired *t*-tests were used to compare individual groups with the documented control.

## Results

### 5-week treatment with mixtures of mouse or human immune Ig and anti-idiotype Ig, attenuates OVA-induced IgE and IL-4 production

**Fig. 2:**
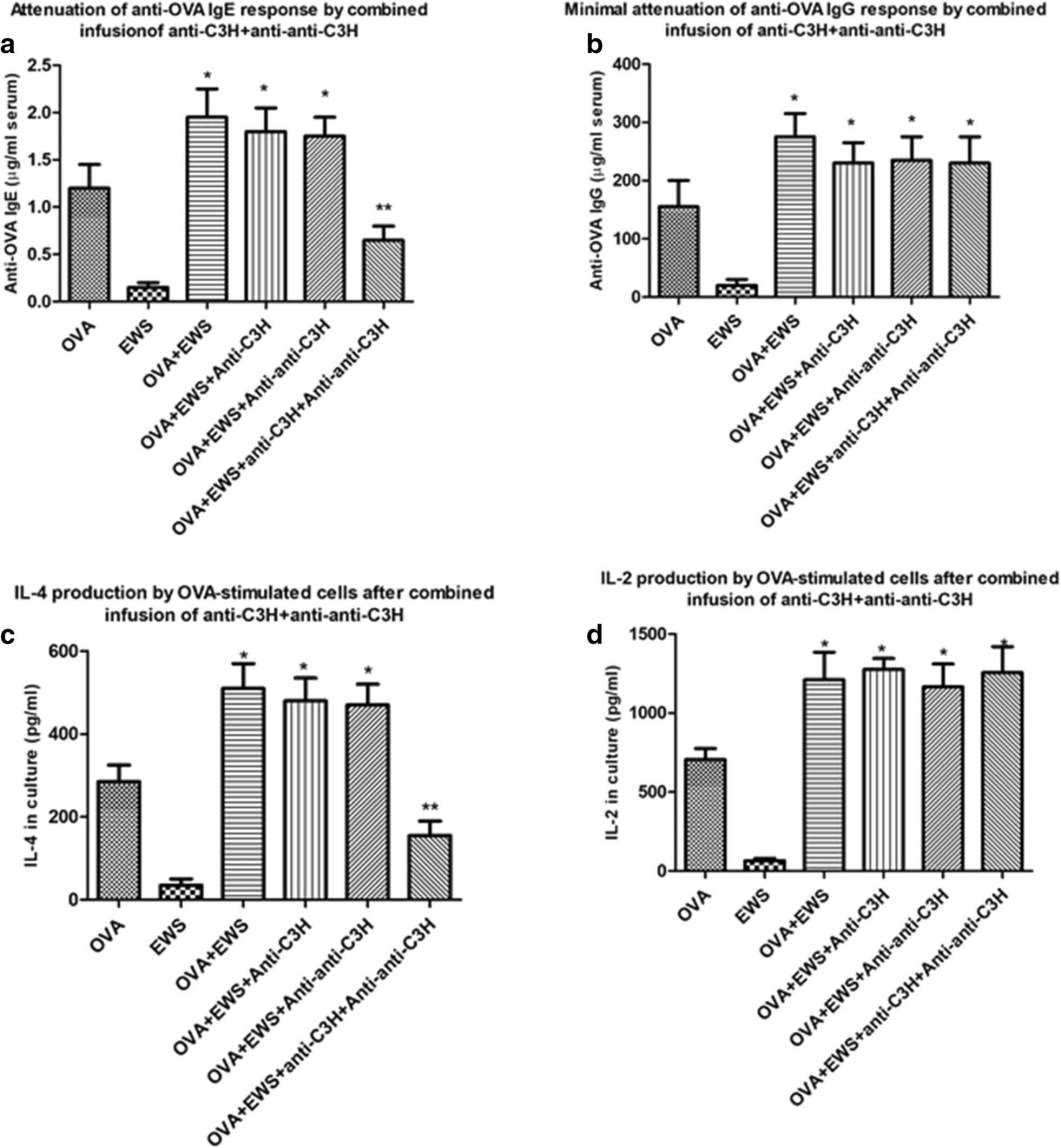
Attenuation of OVA-specific IgE and OVA-induced IL-4 (**a**, **c**) but not IgG and OVA-induced IL-2 (**b**, **d**) in groups of naïve 8 BALB/c mice immunized with OVA in alum (see “Materials and methods” and Fig. 1a) and in addition receiving anti-C3H immune serum alone, anti–anti-C3H serum alone, or a combination of the two antibodies at weekly intervals (×5 injections). All mice received egg white solution (EWS) in their drinking supply. Data show mean ± SD of IgE/IgG levels in serum, or 72 h-induced cytokines in splenocyte cultures in animals receiving a final boost of OVA in alum 7 days after the final immunoglobulin treatment and sacrificed 10 days later. The OVA alone group of mice received normal mouse serum iv. The EWS group received no immunization with OVA in alum. *p < 0.05 compared with mice receiving normal mouse serum (OVA); **p < 0.05 compared with OVA + EWS group

In our first study we confirmed our previous report that treatment of BALB/c mice with a mixture of antibodies designed to stimulate idiotype: anti-idiotypic interactions could attenuate induction of an OVA Th2 response, as assessed by serum IgE levels or antigen-induced IL-4 production in vitro [19]. Naïve groups of 8 mice received Igs given im as indicated in “Materials and methods” (Fig. 1a), beginning 7 days after the first immunization with OVA in alum. 7 days following 5 Ig infusions mice received a final OVA-alum immunization, with sacrifice 10 days later. OVA-specific serum IgE/IgG was measured by ELISA (Fig. 2a, b), and OVA-induced IL-2/IL-4 measured by ELISA at 72 h from spleen cultures (Fig. 2c, d). Only mice receiving both Igs showed a significant decrease in induction of IgE (Fig. 2a) and IL-4 production (Fig. 2c). No attenuation of OVA-specific IgG or IL-2 production was seen (Fig. 2b, d), arguing against the effect being one of general immunosuppression, but more a regulation of a Th2 dependent immune response. Consistent with this, further studies showed a similar suppression of OVA-induced IL-13 production was seen in vitro from cells of the same mice (Additional file 1: Figure S1A), and that attenuation of OVA-IgE production and OVA-induced IL-4/IL-13, but not IL-2, were strongly correlated while that of OVA-induced IgE production and IL-2, but not IL-4/IL-13 were also correlated (Additional file 1: Figure S1B).

**Fig. 3:**
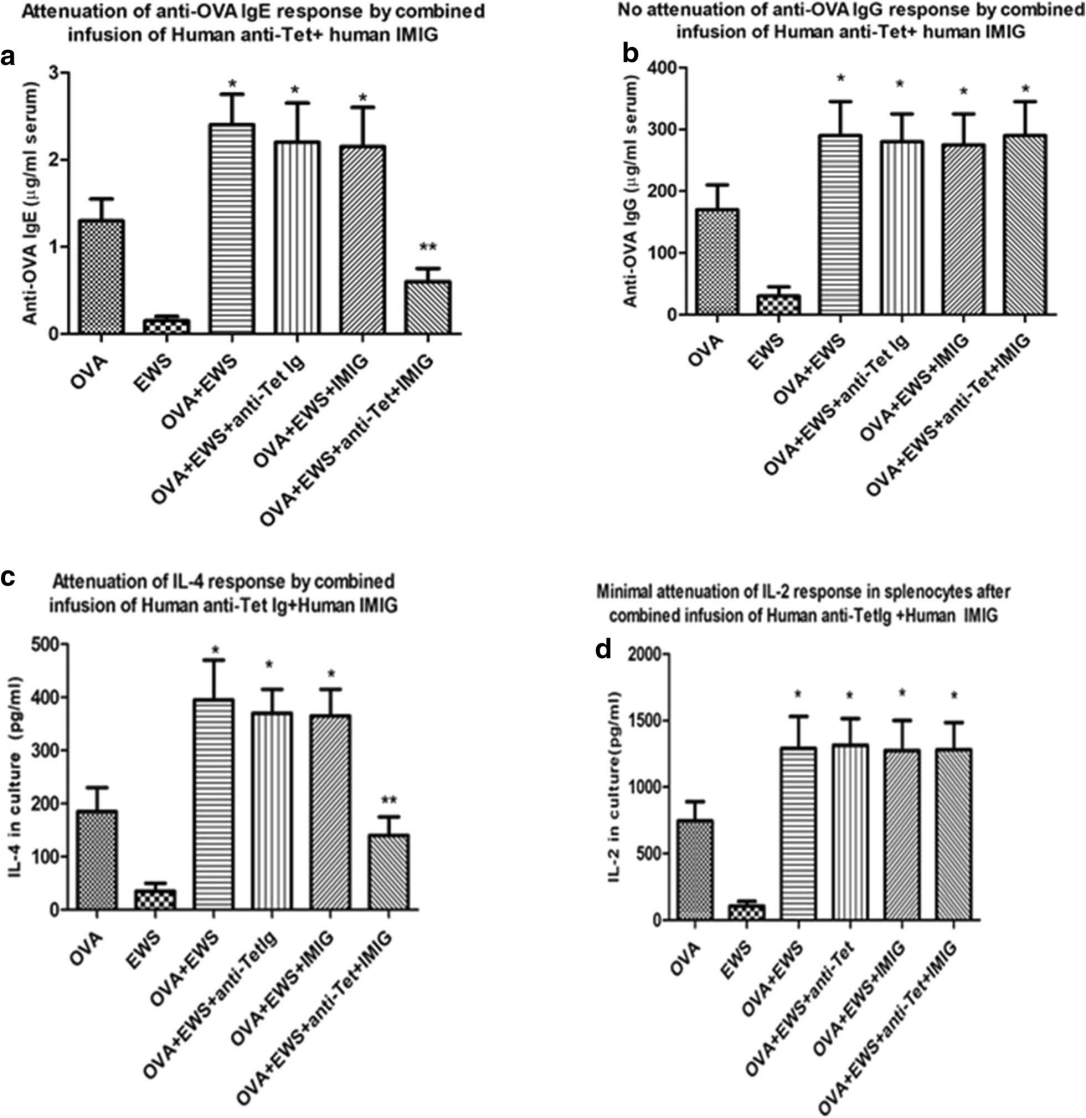
As for Fig. 2, but now showing attenuation of OVA-specific IgE and OVA-induced IL-4 (**a**, **c**) but not IgG and OVA-induced IL-2 (**b**, **d**) in groups of naïve 8 BALB/c mice immunized with OVA in alum (see “Materials and methods”) and in addition receiving pooled human anti-Tet immune Ig serum alone (10 μg/mouse), human IMIG alone (75 μg/mouse), or a combination of the two antibodies at weekly intervals (×5 injections). *p < 0.05 compared with mice receiving normal mouse serum (OVA); **p < 0.05 compared with OVA + EWS group

Based on the theoretical concept that the combined Ig treatments given produced the immunoregulation seen through perturbation of a T (and B) cell immune idiotypic network in the host animals, we had also predicted that the combined anti-idiotype and immune Igs need not be derived from the same vertebrate species [19, 20]. In a recent manuscript we have indeed reported that pooled Human immune Ig (anti-Tet) and pooled human anti-idiotype Ig, using commercial IVIG [27] given intramuscularly, hence IMIG, as a source, could lead to desensitization of allergic response in dogs and mice [23]. Data in Fig. 3 further support this claim and show that human IMIG and anti-Tet Ig produce an equivalent attenuation of IgE and IL-4 production as the murine counterparts (compare with Fig. 2) in naïve BALB/c mice. Note that testing of serum from mice receiving human Ig over the 5-week period shown revealed no detectable anti-human Ig reactivity as assayed with ELISA plates coated with human Ig and developing assays with HRP-coupled anti-mouse Ig (Additional file 2: Figure S2). This was not unexpected given the low doses of human Ig given, and the absence of adjuvant used.

In subsequent studies we found that a similar attenuation of IgE and IL-4 was seen in mice receiving both Igs over a broad dose range (Additional file 3: Figure S3, panels A–D). Note when either anti-Tet or IMIG were used at lower doses suppression was less prominent, though suppression waned faster at reduced doses of IMIG rather than anti-Tet. We hypothesize this reflects a greater concentration of functional immune anti-Tet Ig in the sample used than of anti-idiotype Ig in the polyclonal IMIG used, which was still effective at 50 μg/mouse at all anti-Tet doses. Without use of purified titrated dosing of each reagent this remains unproven. We have also shown that in mice receiving the Igs as a subcutaneous injection at weekly intervals suppression is still seen (Additional file 4: Figure S4, panels A–F). Note that in these latter studies we showed significant attenuation of IL-31 cytokine production also.

### Attenuation of IgE production, and of OVA induce IL-4 release, persists > 14 weeks post Ig treatment with anti-idiotype and immune Ig

**Fig. 4:**
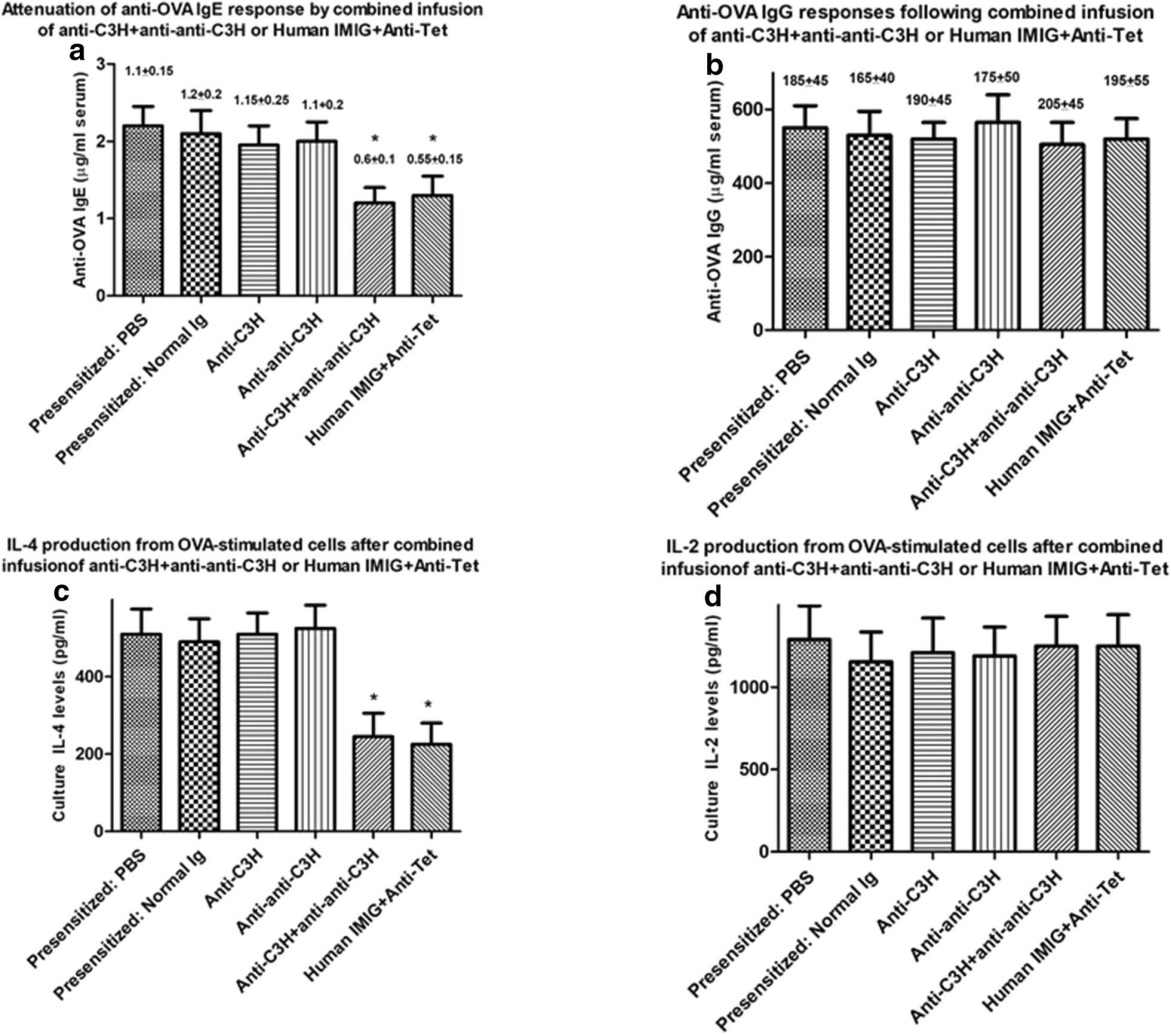
Persistent attenuation of OVA-specific IgE and OVA-induced IL-4 (**a**, **c**) but not IgG and OVA-induced IL-2 (**b**, **d**) in groups of 8 BALB/c mice immunized with OVA in alum (see “Materials and methods”) and in addition receiving the immunoglobulins shown in Figs. 2 and 3 at weekly intervals (×5 injections). All OVA immunized mice were given EWS to drink from day 7 post the first immunization. All mice received boosts of OVA as in Figs. 2 and 3, with a small sample of serum assayed for IgE and IgG 7 days later (figures in parentheses). Mice were rested for a further 11 weeks before receiving a final boost of OVA in alum with sacrifice 10 days later. Data show mean ± SD of IgE/IgG levels in serum in mice at > 14 weeks post the last immunoglobulin treatment, or cytokine levels in 72 cultures of OVA-stimulated splenocytes from these mice. Two control groups (far left in each panel) received either PBS and OVA immunization only, or OVA immunization and normal mouse Ig (not anti-idiotype and immune Ig) respectively. *p < 0.05 compared with mice receiving normal mouse serum and OVA

In order to assess the longevity of the suppression of IgE and IL-4 production seen following the 5-week Ig treatment given in Figs. 1 and 2 we performed the following study. Mice received OVA immunization as before, with 5 weekly im injections of different combinations of anti-C3H Ig and anti–anti-C3H Ig, or human IMIG and anti-tet. Following the final Ig treatment mice were boosted with OVA in alum and a test bleed obtained from the tail (50 μl) 10 days later (as in Fig. 1a but without sacrifice of mice at day 52). OVA-specific IgE and IgG were measured by ELISA (figures in parentheses above histograms in panels a, b of Fig. 4). All mice were rested (but maintained on EWS) for 11 weeks (to day 129) and then received a final boost of OVA in alum, with sacrifice and harvest of splenocytes and serum 10 days later (day 139). Again OVA-specific IgE and IgG were assayed (Fig. 4a, b) and IL-4 and IL-2 produced by OVA stimulated splenocytes in ELISA measured at 72 h (Fig. 4c, d).

These data show clearly that the attenuation of IgE and IL-4 production following a short (5 week) course of combined anti-idiotype + immune Ig, using either homologous (mouse) or heterologous (human) immunoglobulin, was maintained in the absence of further Ig injections, for at least 14 weeks post injection. This has significant implications for the utility of such a protocol for clinical application.

### Use of combined Ig treatment to desensitize OVA-immune animals as judged by serum IgE levels and OVA-induced IL-4 production

**Fig. 5:**
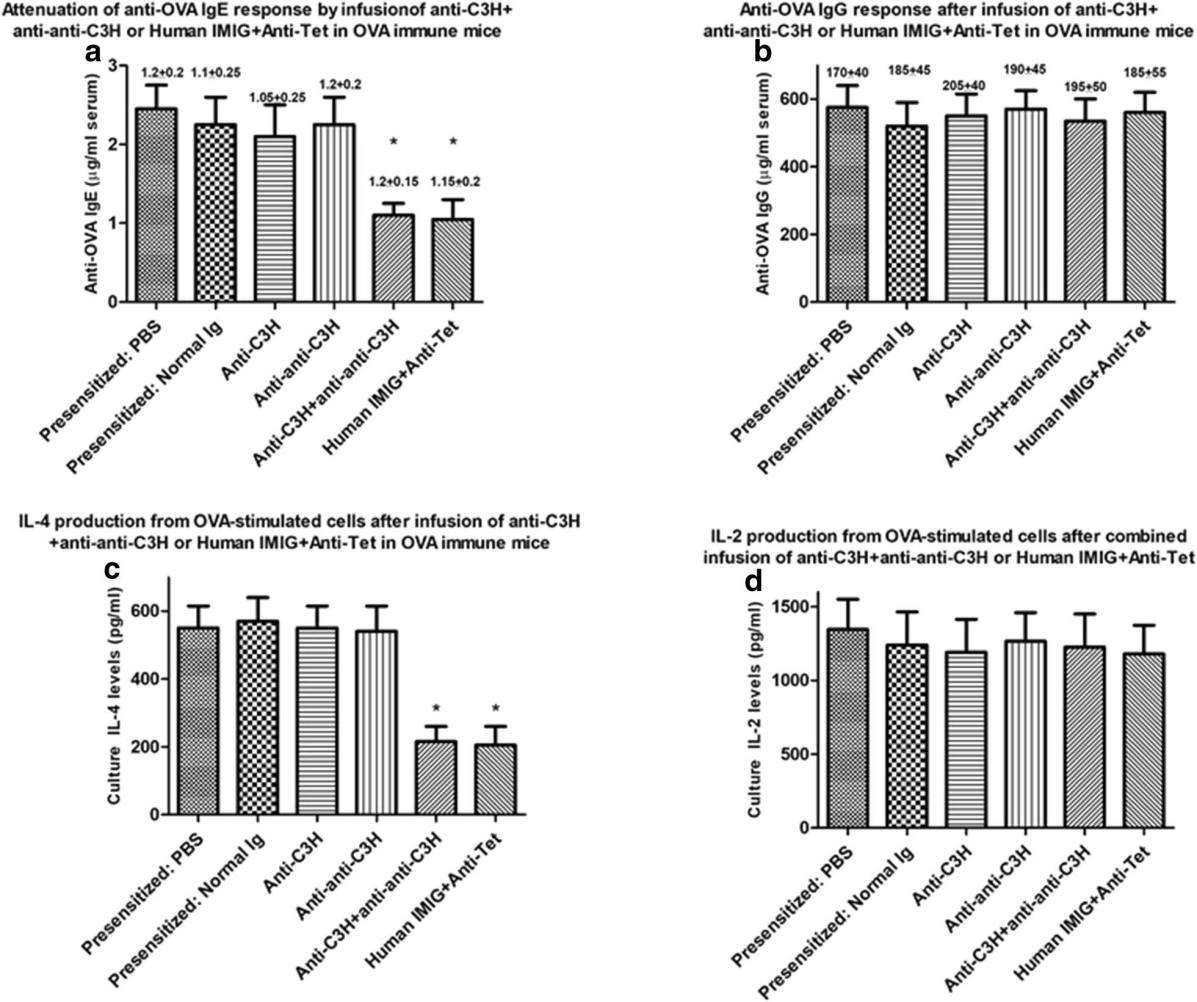
Attenuation of OVA-specific IgE or OVA-induced IL-4 (**a**, **c**) but not IgG or OVA-induced IL-2 (**b**, **d**) responses in groups of 8 BALB/c mice *pre*-*immunized* over a 7 week period with OVA in alum (see text, “Materials and methods” and Fig. 1b) before receiving anti-C3H immune serum, anti–anti-C3H serum, or a combination of the two antibodies at weekly intervals (×5 injections), or a combination of human IMIG and anti-Tet. All OVA immunized mice were given EWS to drink from day 7 post the first immunization. Data show mean ± SD of Ig levels in serum in mice receiving a final boost of OVA in alum 2 days after the final immunoglobulin treatment and sacrificed 10 days later, or cytokine levels in 72 cultures of OVA-stimulated splenocytes from these mice (Fig. 1b). Data in parentheses above each bar indicate the OVA-specific serum IgE or IgG levels in mice immediately after sensitization and before commencing treatment with anti-C3H immune Ig and/or anti–anti-C3H Ig, or human IMIG + anti-Tet. Control groups of immune mice received treatment with PBS or normal mouse Ig. *p < 0.05 compared with all other groups of mice

In order to assess whether application of a similar combined Ig protocol could desensitize mice after first sensitizing to OVA in alum, we used similar studies to Fig. 1a but using a group of mice pre-immunized with OVA (as indicated in Fig. 1b). A sample of serum (50 μl) was obtained from the tail vein for all mice for measurement of OVA-specific serum IgE or IgG by ELISA at day 52 (see Fig. 1b). Mice were randomly to groups of 8 which then received weekly treatment with C3H anti-BL/6 Ig, anti–anti-C3H Ig, or both immunoglobulins in different limbs, or a combination of Human IMIG and anti-Tet (Fig. 1b). Control mice received normal C3H Ig only or PBS only. All mice received a further boost of OVA in alum 2 days after the last immunoglobulin treatment and were sacrificed 10 days later to assess both OVA-specific serum IgE/IgG, and OVA-induced IL-2/IL-4 from splenocytes re-challenged in vitro with OVA for 72 h, as in Figs. 2, 3, and 4. Data for one of two studies are shown in Fig. 5.

It is clear from comparison of Figs. 2, 3 and 5 that the combination of anti-C3H Ig and anti–anti-C3H Ig, as well as Human IMIG + anti-Tet, was effective in attenuating both OVA-serum IgE responses (panels A of Figs. 2, 3 and 5) and OVA-induced IL-4 responses (panels c of the same figures) in either naïve (Figs. 2, 3) or pre-immune (Fig. 5) mice. Note that in the pre-immune mice, combination immunoglobulin treatment resulted in levels of OVA-specific IgE after treatment which were less than levels seen immediately after sensitization with OVA (figures in parentheses) but before treatment with combined Ig. A similar attenuation of IL-4 production compared to levels seen after sensitization with OVA also was seen in immune mice receiving treatment with combined Ig before final stimulation in culture (compare panels c in these figures). Note that as controls, levels of OVA-specific IgG, and levels of OVA-induced IL-2 production, were unaffected by the treatment used (see panels b and d in Figs. 2, 3, 5). Thus, our data indicate that the intervention with combined anti-idiotype and immune Ig is not causing a generalized immunosuppression in treated animals, but an “immune deviation” away from Th2 dependent antigen-specific IgE and IL-4 production.

### Evidence that CD4^+^ and CD8^+^T cells regulate OVA-specific Th2 responses induced through perturbation of idiotypic networks using Bl/6 anti-C3H Ig and anti–anti-C3H Ig

**Fig. 6:**
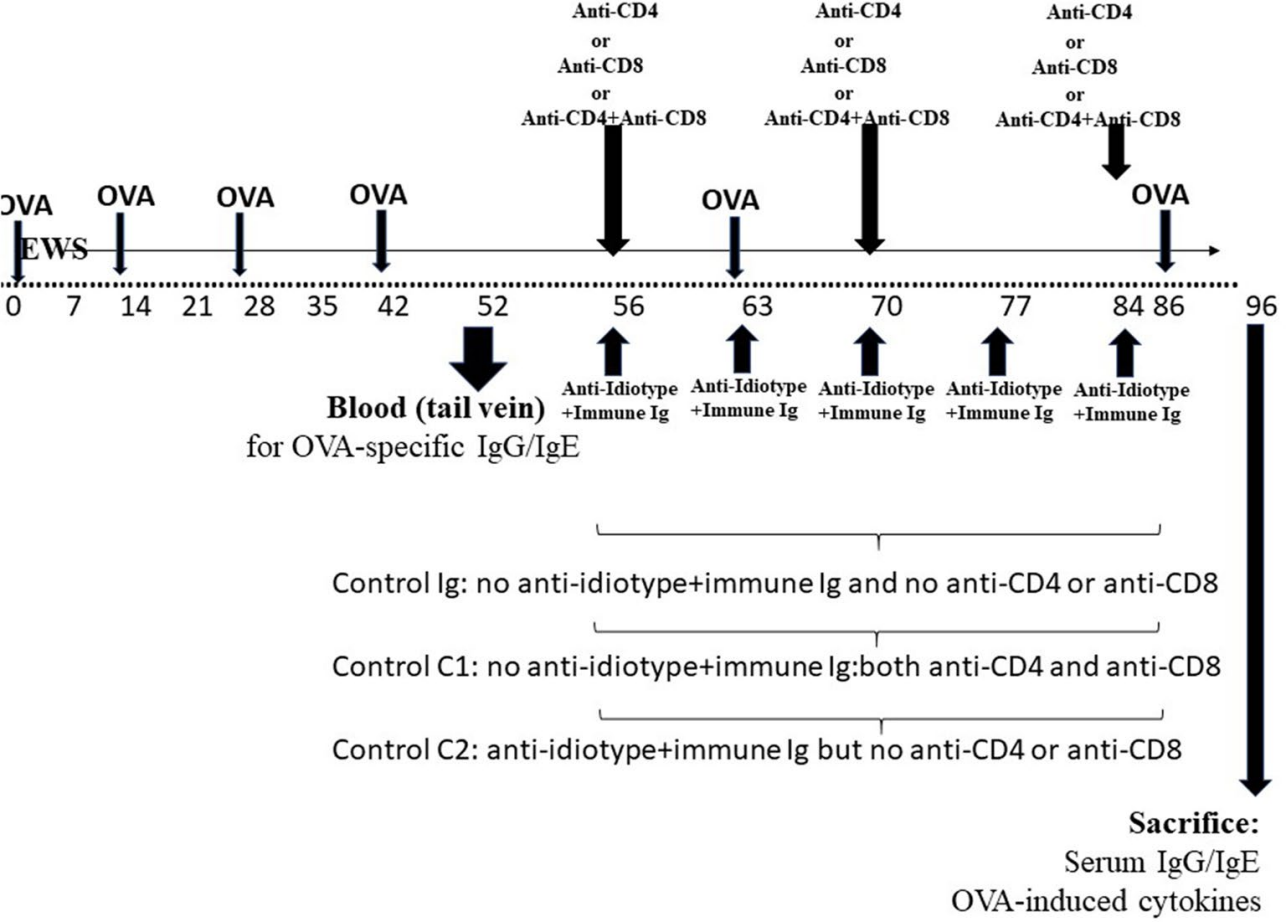
Schematic to show sequence of delivery of iv anti-CD4, anti-CD8 or combined anti-CD4^+^ anti-CD8 in mice preimmunized with OVA in alum (given ip) and receiving anti-idiotype Ig and immune Ig (both given im at separate sites). All mice received egg white solution (EWS) in their drinking supply from the time shown (day 7) throughout the course of study. Animals were sacrificed for measure of OVA-specific IgG and IgE and OVA-induced cytokines in 72 h cultures of splenocytes. Tail bleeds were used at 52 days to assess induction of OVA-IgG and IgE, and animals were sacrificed for measure of OVA-specific IgG and IgE and OVA-induced cytokines in 72 h cultures of splenocytes 10 days after a final OVA boost (day 96)

**Fig. 7:**
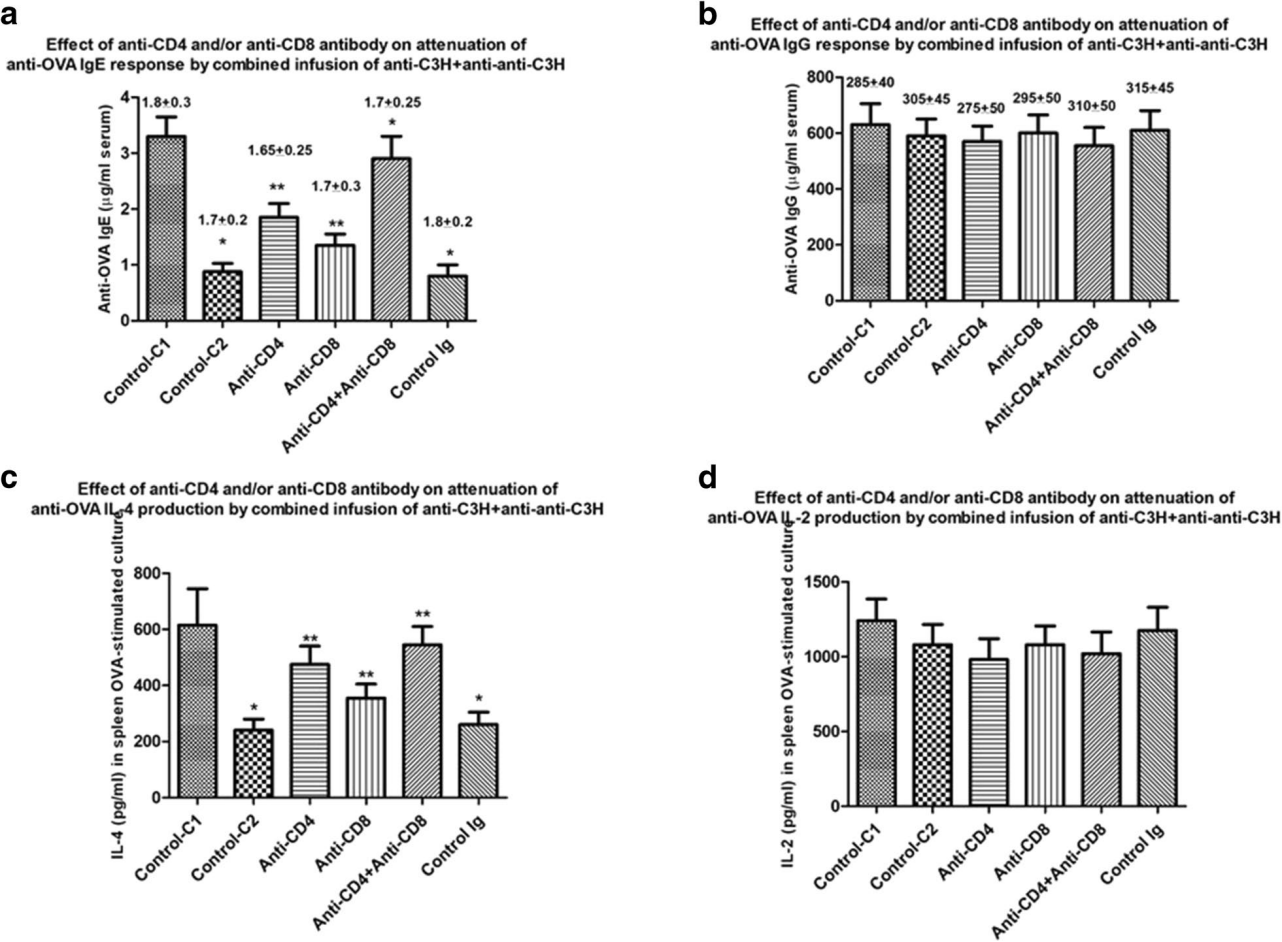
Amelioration by T depletion of attenuation of OVA-specific IgE and OVA-induced IL-4 production (**a**, **c**) but not IgG or OVA-induced IL-2 production (**b**, **d**) in groups of 8 BALB/c mice pre-immunized with OVA in alum (see text and “Materials and methods”), and subsequently receiving 5 weekly treatments with combined mouse immune Ig (anti-C3H) and anti-idiotype Ig (anti–anti-C3H). A control group (Control C1) received no further treatment after OVA immunization. Subgroups of 7 mice/group received either no further treatment besides the 5 combined mouse Ig treatments (Control C2), or treatment with CD4 or CD8 depleting antibodies, or both, at the 1st, 3rd and 5th Ig treatments (see Fig. 6). An additional control group received normal mouse Ig (Control Ig). Data show mean ± SD of Ig levels in serum in mice receiving a final boost of OVA in alum 7 days after the final immunoglobulin treatment and sacrificed 10 days later, or of cytokines in 72 h cultures of splenocytes from mice at sacrifice. Data in parentheses above each bar indicate the OVA-specific serum IgE or IgG levels in mice immediately after sensitization and before commencing treatment with anti-C3H immune Ig and/or anti–anti-C3H Ig. *p < 0.05 compared with Control C1. **p < 0.05 compared with Control C2

**Fig. 8:**
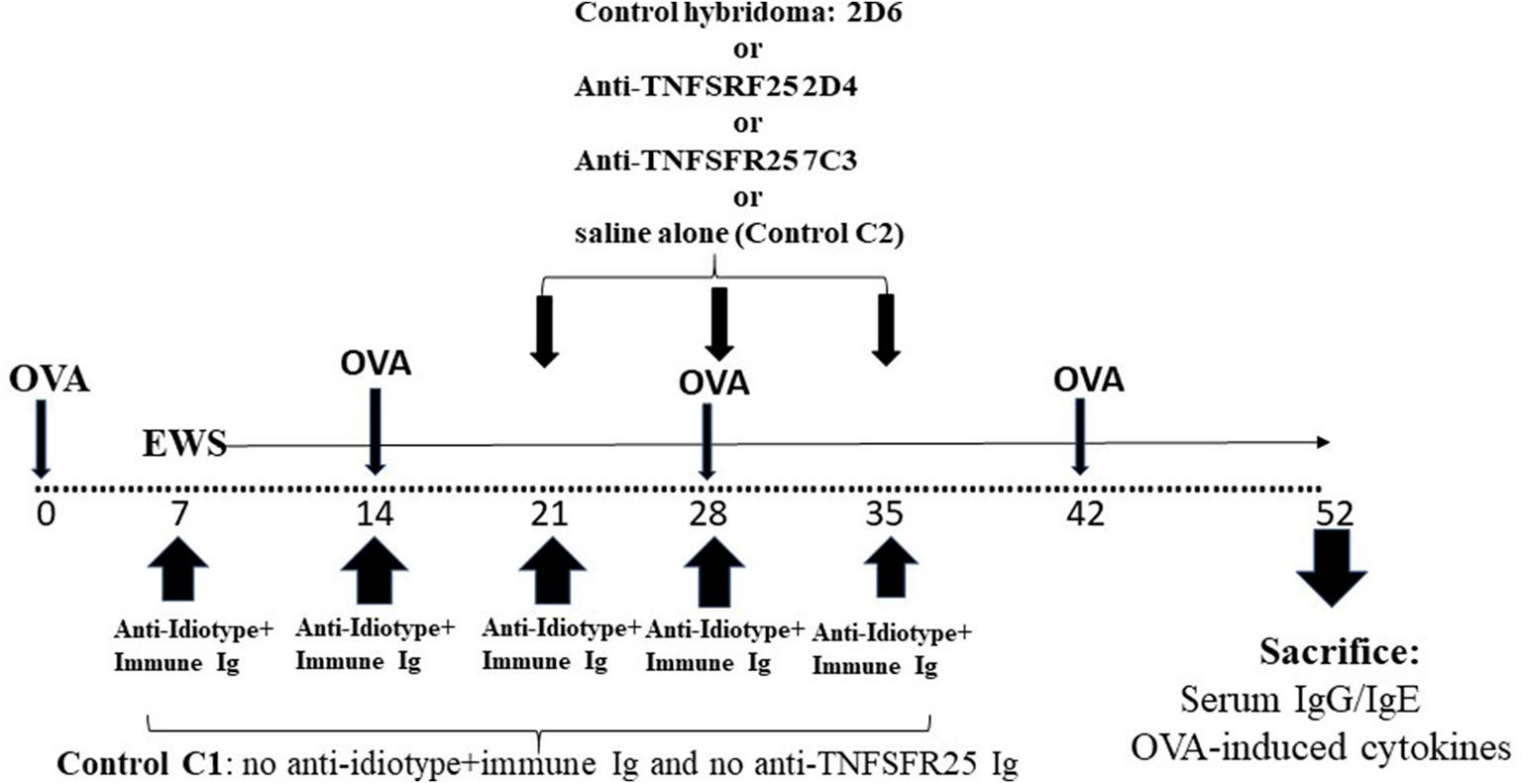
Schematic to show sequence of immunization with OVA in alum (given ip) and anti-idiotype Ig and immune Ig (both given im at separate sites) in naïve mice in combination with iv infusion of rat Ig hybridomas (control, 2D6, or anti-TNFSFR25, 2D4 and 7C3). All mice received egg white solution (EWS) in their drinking supply from the time shown (day 7). Animals were sacrificed as shown for measure of OVA-specific IgG and IgE and OVA-induced cytokines in 72 h cultures of splenocytes

In our previous report we had hypothesized that combinations of BL/6 anti-C3H Ig and anti–anti-C3H Ig led to attenuation of immune responses through mechanisms which included altered regulatory T cell idiotypic networks, as initially postulated by many groups and reviewed elsewhere [7, 13, 14, 19–21]. In order to assess whether similar mechanisms were at least in part implicated in the desensitization of OVA-immune mice described in Figs. 2, 3, 4, and 5 we performed the following study. A group of 42 BALB/c mice were immunized with OVA in alum following the protocol shown in Fig. 6, with ongoing EWS exposure from 7 days. 6 weeks later (day 42) mice received a further boost of OVA in alum. Following tail vein sampling of all mice, a control group of 7 mice subsequently received 5 weekly injections of normal C3H Ig (Control C1 in Figs. 7, 8). The remaining 35 animals were randomly assigned to 5 groups of 7 receiving 5 treatments with C3H anti-BL/6 Ig and anti–anti-C3H Ig. These groups received (iv) in addition either 25 μg anti-mouse CD4, anti-mouse CD8, both anti-CD4 and anti-CD8 (Fig. 6). A control group received no anti-CD4/-CD8 (Control C2 in Figs. 7, 8). Following a last boost of OVA all mice were sacrificed to assess both OVA-specific serum IgE/IgG, and OVA-induced IL-2/IL-4. Data for one of two studies are shown in Fig. 7. Data in Table 1 indicates the % surviving CD4^+^/CD8^+^ cells in all mice at sacrifice (12 days post delivery of the last T depleting sera) and shows clearly the effectiveness of the anti-CD4/anti-CD8 antibody treatments on depletion of the respective T cell subpopulation depletion. Note, however, that dual staining of CD4^+^CD25^+^ and CD8^+^CD25^+^ cells was not performed on all mice at sacrifice.

**Table 1:** Surviving CD4^+^/CD8^+^ cells in splenocytes of mice shown in Figs. 6 and 7

Group of mice	^a^ %CD4^+ b^	% CD8^+ b^
Control Ig	23 ± 4.1	7.1 ± 2.2
Control C1	6.3 ± 1.5*	2.5 ± 0.6*
Control C2	26 ± 3.8	7.0 ± 2.5
Anti-CD4 treated	6.0 ± 2.1*	10 ± 2.9
Anti-CD8 treated	27 ± 5.5	1.9 ± 0.5*
Anti-CD4^+^ anti-CD8 treated	6.7 ± 2.8*	2.0 ± 1.2*

^a^Mice were immunized with OVA; received additional treatment with combined anti-idiotype and immune Ig; and also, anti-CD4 and/or anti-CD8 as indicated in Figs. 6 and 7

^b^% positive CD4^+^ and CD8^+^ cells in splenocytes of mice treated as in Fig. 6 and sacrificed 10 days after the last OVA immunization. Data show means (± SD) for groups of 7 mice

* p < 0.05 compared with first row, control Ig group (no anti-idiotype Ig/Immune Ig and no anti-CD4/-CD8)

Comparison of Control C1 vs Control C2 in Fig. 7 confirms the findings in Fig. 5, that in pre-immune mice combined treatment with C3H anti-BL/6 Ig and anti–anti-C3H Ig led to attenuation of OVA-induced IgE (not IgG) and IL-4 (not IL-2) production. Animals treated in addition by infusion of control mouse Ig iv on the 1st, 3rd and 5th of these combined Ig treatments similarly showed attenuated IgE and IL-4 responses (far right-hand bar in Fig. 7a, c). Mice receiving additional infusions of anti-CD4 or anti-CD8 antibody while undergoing weekly im treatment with C3H anti-BL/6 Ig and anti–anti-C3H Ig showed less suppression of OVA-IgE and IL-4 production than similar mice in control C2 receiving anti-idiotype/immune Ig but no anti-CD4/-CD8. The combination of both anti-CD4 and anti-CD8 treatment led to the greatest attenuation of IgE and IL-4 (see Fig. 7). We conclude that suppression of a Th2 dependent IgE and IL-4 production on OVA-immune mice afforded by weekly treatment with C3H anti-BL/6 Ig and anti–anti-C3H Ig is dependent upon both CD4^+^ and CD8^+^ cells, as we had previously postulated [19].

### Use of anti-TNFRS25 to enhance attenuation of IgE or IL-4 production in mice receiving combined Ig treatment

We [25, 26] and others [22, 28] have suggested that antibodies made against a molecule, TNFRSF25, expressed at the cell surface of CD4^+^CD25^+^ Tregs may augment immunoregulation by putative Tregs in vivo-at least one such study has reported this molecule enhanced regulation of allergic immunity [22]. We have reported on production of several rat mAbs to a peptide of TNFRSF25 common to both the human and mouse molecules and observed by FACS and ELISA (with plate-bound cells) significant binding of several mAbs to enriched CD4^+^Tregs. mAbs binding CD4^+^Tregs produced functional expansion of murine Tregs in vitro, as assayed in MLCs by an augmented ability to attenuate induction of CTL and proliferation in vitro, in the absence of any evidence for direct cytotoxicity to CD8^+^ CTL or CD4^+^ cells proliferating in MLCs [26]. Moreover, infusion of these mAbs in vivo into mice receiving skin allografts along with additional manipulations designed to augment a role for Treg in increased graft survival showed improved (~ twofold) long-term graft survival [25]. As an additional study to assess a possible role for CD4^+^ Tregs in attenuation of OVA-induced IgE and IL-4 production in mice receiving combined treatment with C3H anti-BL/6 Ig and anti–anti-C3H Ig we performed the following study.

**Fig. 9:**
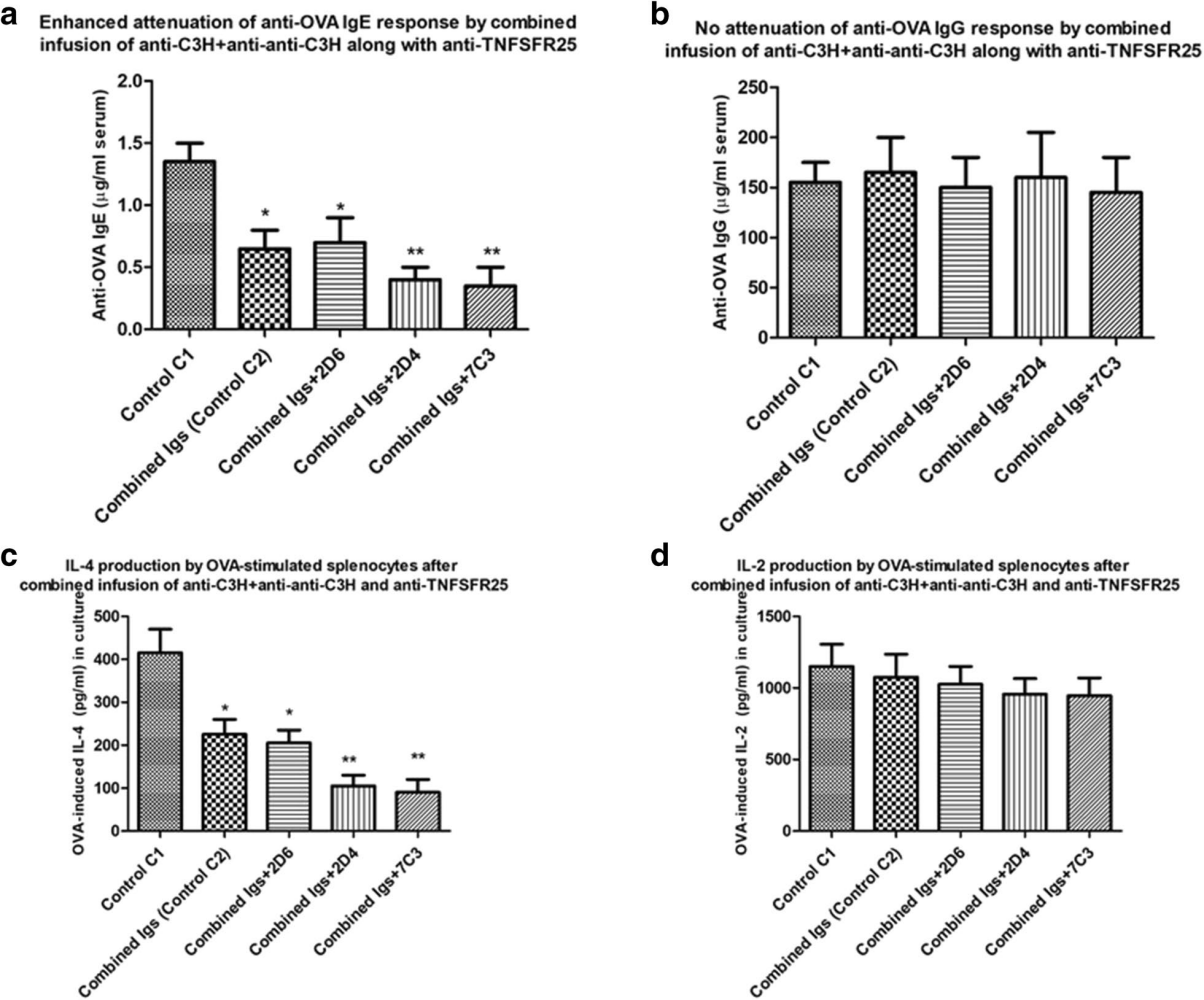
Enhanced attenuation of OVA-specific IgE and OVA-induced IL-4 production (**a**, **c**) but not of IgG or OVA-induced IL-2 production (**b**, **d**) in groups of 5 BALB/c mice immunized with OVA in alum and receiving combined anti-C3H and anti–anti-C3H Ig as in Fig. 2, and in addition receiving further infusions of rat mAbs directed to TNFSFR25 [25, 26]. A control group, Control C1, received no combined immune Ig/anti-idiotype Ig treatment. Other groups of mice received iv infusions of 10 μg rat Ig prepared from a control hybridoma supernatant (2D6) or similar quantities of Ig purified from anti-TNFSFR25 hybridomas (2D4, 7C3) on the last 3 weeks of treatment with the anti-C3H and anti–anti-C3H Ig. A control group, control C2, received no iv infusions. Data show mean ± SD of IgE and IgG levels in serum, and IL-2/IL-4 in 72 h cultures of OVA-stimulated cells, of mice receiving a final boost of OVA in alum 7 days after the final immunoglobulin treatment and sacrificed 10 days later. *p < 0.05 compared with Control C1; **p < 0.05 compared with Control C2 and group with 2D6

Groups of 5 naïve BALB/c mice (25 in total) were immunized with OVA in alum as in Figs. 1a, 2 and 8 with exposure to EWS from day 7. 20 mice received treatment with C3H anti-BL/6 Ig and anti–anti-C3H Ig as in Fig. 8. A control group (C1) received no other treatment. 3 groups of 5 mice/group received in addition 3 iv infusions at 7 days intervals of either control rat Ig (10 μg/mouse purified from hybridoma 2D6), or 10 μg/mouse of previously described 2D4 or 7C3 anti-TNFSRF25 mAbs—see ([26] and Fig. 8). An additional control group (Control C2) received no iv Ig treatment. All mice received a boost with OVA with sacrifice 10 days later as shown in Fig. 8. Data from one such study (of 2) are shown in Fig. 9.

Comparison of Controls C1 and C2 in Fig. 9 confirms previous data showing attenuation of IgE and IL-4 production in mice receiving combined infusion with C3H anti-BL/6 Ig and anti–anti-C3H Ig (panels A/C). Importantly, while this attenuation was unaffected by additional iv infusion of a control rat Ig (from hybridoma 2D6)-see * in groups C2 and 2D6 in Fig. 9a, c, infusion of Ig purified from the anti-TNFSFR25 mAbs 2D4 or 7C3 enhanced the attenuation seen (** in Fig. 9a, c). Again, there was no significant effect seen at the level of IgG responses or OVA-induced IL-2 production-see panels B/D in Fig. 9. Note however, we did not measure directly the recovered levels of CD4^+^CD25^+^ or CD8^+^CD25^+^ cells by FACS on sacrifice of mice in the various groups shown in Figs. 8 and 9.

## Discussion

Exploration of novel therapies for treatment of allergic diseases have highlighted measures which decrease IgE levels, and target Th2 cytokines (e.g. IL-4, IL-5, IL-31) thought to be implicated in control of those levels and other cell populations (eosinophils/basophils) implicated in allergic disease [29–33]. In addition, concerted efforts have ben made to improve the efficacy of allergen-induced attenuation of responses [7, 9–12, 15–18]. We have previously reported on the use of a novel therapy, aimed at manipulating self-reactive immune regulatory networks through deliberate perturbation of idiotype: anti-idiotype interactions, as a potential mechanism to attenuate several pathological immune reactions in rodents, including allergic sensitization to ovalbumin [19]. In the discussion of our results from this previous analysis, we hypothesized that the attenuation of mouse Th2 responses to ovalbumin which following repeated injection with immune Ig and anti–anti-self Ig (see also Figs. 1a, 2) were best understood in terms of an effect mediated by a re-setting of both B cell and T regulatory cell immune networks in mice receiving combined immune Ig and anti-idiotype Ig [19–21]. A corollary of this hypothesis was that in vertebrates with a shared ancestral immune system, even heterologous Igs (immune + anti-idiotype) would produce the same effects [20]. Data in Fig. 3 confirm that indeed in BALB/c mice sensitized with ovalbumin, attenuation of allergic responses was seen following treatment with commercial immune Ig (anti-Tet) and anti-idiotype Ig of human origin (using commercial IVIG as a source of anti-idiotype [27].

In terms of the value of the approach described in a clinical setting it was important to address the issues of whether attenuation of allergy was seen only as a preventative strategy, as shown to date ([19]; Figs. 2, 3), or also as a therapeutic strategy in already previously sensitized individuals. In addition, it was important to assess the duration of attenuation of allergic responses seen using the protocol described. In this regard the data of Fig. 3 are 4 of great importance. Figure 4 indicates that in the mouse model shown (Fig. 1a), even in the absence of any further desensitization treatments with combined Igs, and despite ongoing exposure to allergen (EWS in the drinking supply), mice maintain a decreased IgE and OVA-induced IL-4 production for > 14 weeks following the last Ig treatments. Figure 5 shows equally unequivocally that desensitization is seen under therapeutic circumstances, in mice which received treatment with immune Ig+ anti-idiotype Ig commencing only after first developing a documented Th2 response with high IgE levels (see Fig. 1b). It is worth stressing at this stage that the therapy used is one which occurs through mechanisms independent of the antigen used for sensitization, a feature of significant importance clinically when often the allergen cannot be identified and/or synthesized for allergen-specific desensitization [15–18].

In terms of identifying the mechanism(s) involved in regulation induced by the combined immune Ig and anti-idiotype Ig, the data in Figs. 6 and 7 and Figs. 8 and 9 argue strongly in favour of an important role for both CD4^+^ and CD8^+^ T cells in regulation. There is already abundant data to support such a role for T cells in allergic diseases [7, 13, 14, 22, 33], and we show that T depletion, particularly of both CD4^+^ and CD8^+^ cells following the protocol described in Fig. 6 abolishes the ability of the Ig treatment used to suppress Th2 immunity, in keeping with decreased numbers of CD4^+^ and/or CD8^+^ cells recovered from such mice. T depleting antibodies alone in the absence of anti-idiotype and immune Ig (control C1 group, Figs. 6, 7), has no effect on IgE and IL-4 levels (compare control C1 with control Ig and control C2 groups in Fig. 7). Interestingly, and further supporting a previously hypothesized role for TNFRSF25 Tcells in the suppression, we found that mAbs which expanded such cell populations [25, 26] further enhanced suppression of Th2 immunity (Figs. 8, 9). Note that as previously reported [25, 26] the mAbs used in Figs. 8 and 9 did not in themselves induce Tregs directly. It is worthy of note that we have not explored a potential role for other regulatory cells, including Bregs, induced by anti-idiotype + immune Ig treatment. Bregs are a heterogeneous group of B lymphocytes with known immunosuppressive functions [34, 35], which may contribute to immunoregulation through increasing the number of Treg cells [36]. However, in studies in allergy which have supported an important role for B regs [37, 38], some consensus seems to reside in a role for a Th2 cytokine mediated enhancement of Breg IL-10 production as an important mechanism of action-this has not been investigated in the studies described [39].

In summary, we have now confirmed that a previously described novel therapy, using a combination of immune Ig and anti-idiotype Ig, of either homologous or heterologous origin, can induce long-lasting attenuation of Th2 responses, used as a surrogate of responses predisposing to allergy, when used in both a preventative, and more useful clinically, therapeutic regime. Attenuation occurs independent of any relation between the immune Igs/anti-idiotype Igs and the allergen, thus removing the need for definition of the antigen prior to use. Treatment is effective when Igs are given by any of multiple routes (systemic, intravenous), intramuscular or subcutaneous. In unpublished studies we have found significantly reduced efficacy if the two Igs are mixed together and given at a single site, which we take to imply a need for each of the two components to engage the immune system independently, with some neutralization of this action when that engagement occurs in close physical proximity. With our recent report on the use of this therapy to attenuation of skin allergy responses in dogs [23], we suggest that the treatment described represents a potential major advance in clinical management of such diseases.

## Supplementary information

**Additional file 1: Figure S1.** A. As in Figure 2, attenuation of OVA-induced IL-13 in the same groups of naïve 8 BALB/c mice immunized with OVA in alum (Figure 1a) and receiving anti-C3H immune serum alone, anti-anti-C3H serum alone, or a combination of the two antibodies at weekly intervals (5× injections). Data show mean ± SD of 72 h-induced cytokines in splenocyte cultures of animals sacrificed 10 days after a final boost of OVA. All groups were as shown in Figs. 1a and 2. *, p < 0.05 compared with mice receiving normal mouse serum (OVA); ** p < 0.05 compared with OVA+EWS group. B. Correlation analysis (using Prism software) for cytokines (IL-2, IL-4 and IL-13) with either serum OVA-specific IgG levels (upper panel) or OVA-specific IgE levels (lower panel), using data shown in Fig. 2a–d and Additional file 1: Figure S1A. As indicated IgG levels were strongly correlated with IL-2 levels only, while IgE levels were correlated with IL-4/IL-13.

**Additional file 2: Figure S2.** Absence of detectable mouse anti-human IgG responses in mice receiving heterologous (human) Anti-Tet immune Ig, IMIG, or a mixture of the two (at separate sites). 100 μl serum (diluted 1:3) was assayed in duplicate from each of the mice at sacrifice (after 5 injections) shown in Figure 3, with ELISA plates coated with human IgG (100 ng/well), and commercial goat anti-mouse Ig-HRP as developing agent (1:1000). A commercial mouse anti-Human IgG was used as a positive control (ThermoFisher, 1:1000). Data show group means ± SD. The dotted line shows the detection limit in the assay (20 pg/ml).

**Additional file 3: Figure S3.** Comparison of attenuation of OVA-specific immune response (compare with Fig. 3) in mice receiving different doses, ranging from 250 μg/mouse to 10 μg/mouse, of human IMIG or anti-Tet immune Ig given im at weekly intervals. Control groups received the highest dose of IMIG (250 μg/mouse) or an intermediate dose of anti-Tet Ig (50 μg/mouse) alone. Data show mean ± SD of Ig levels in serum, or cytokines at 72 h in culture supernatants. In subsequent studies we have routinely used IMIG (75 μg/mouse) and anti-Tet Ig (10 μg/mouse). *, p < 0.05 compared with mice receiving no Human IMIG or anti-Tet Ig.

**Additional file 4: Figure S4.** Comparison of attenuation of OVA-specific immune response in mice (see Fig. 3) receiving combination treatment with human IMIG or anti-Tet immune Ig each given via either an intramuscular or subcutaneous route of administration at weekly intervals. Control groups received either the IMIG or anti-Tet alone, again via either of these two routes. Data show mean ± SD of Ig levels in serum, or cytokines at 72 h in culture supernatants. Note that cultures in this instance were also assayed for IL-31 and IL-33, given the recent interest in their use as markers of allergic inflammation. *, p < 0.05 compared with mice receiving no Human IMIG or anti-Tet Ig.

## Data Availability

Data and materials (where available) included in this study will be made freely available.
