# Supplementary figures and images for: Mechanism(s) of prolonged attenuation of allergic responses after modulation of idiotypic regulatory network
Source: Allergy Asthma Clin Immunol. 2019 Dec 4;15:79. doi: 10.1186/s13223-019-0393-7 (PMC6892213; doi:10.1186/s13223-019-0393-7)

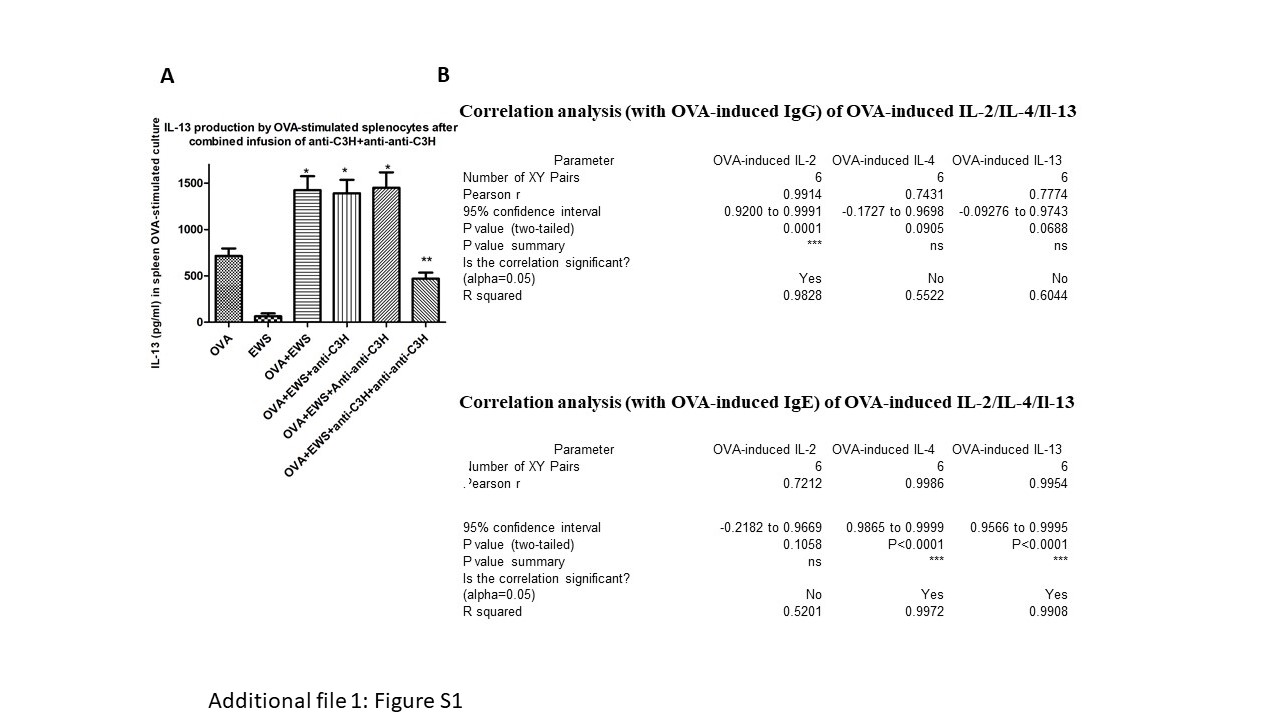

Supplement: Supplementary file 1 — Additional file 1: Figure S1. A. As in Figure 2, attenuation of OVA-induced IL-13 in the same groups of naïve 8 BALB/c mice immunized with OVA in alum (Figure 1a) and receiving anti-C3H immune serum alone, anti-anti-C3H serum alone, or a combination of the two antibodies at weekly intervals (5× injections). Data show mean ± SD of 72 h-induced cytokines in splenocyte cultures of animals sacrificed 10 days after a final boost of OVA. All groups were as shown in Figs. 1a and 2. *, p < 0.05 compared with mice receiving normal mouse serum (OVA); ** p < 0.05 compared with OVA+EWS group. B. Correlation analysis (using Prism software) for cytokines (IL-2, IL-4 and IL-13) with either serum OVA-specific IgG levels (upper panel) or OVA-specific IgE levels (lower panel), using data shown in Fig. 2a–d and Additional file 1: Figure S1A. As indicated IgG levels were strongly correlated with IL-2 levels only, while IgE levels were correlated with IL-4/IL-13. [file 13223_2019_393_MOESM1_ESM.jpg]

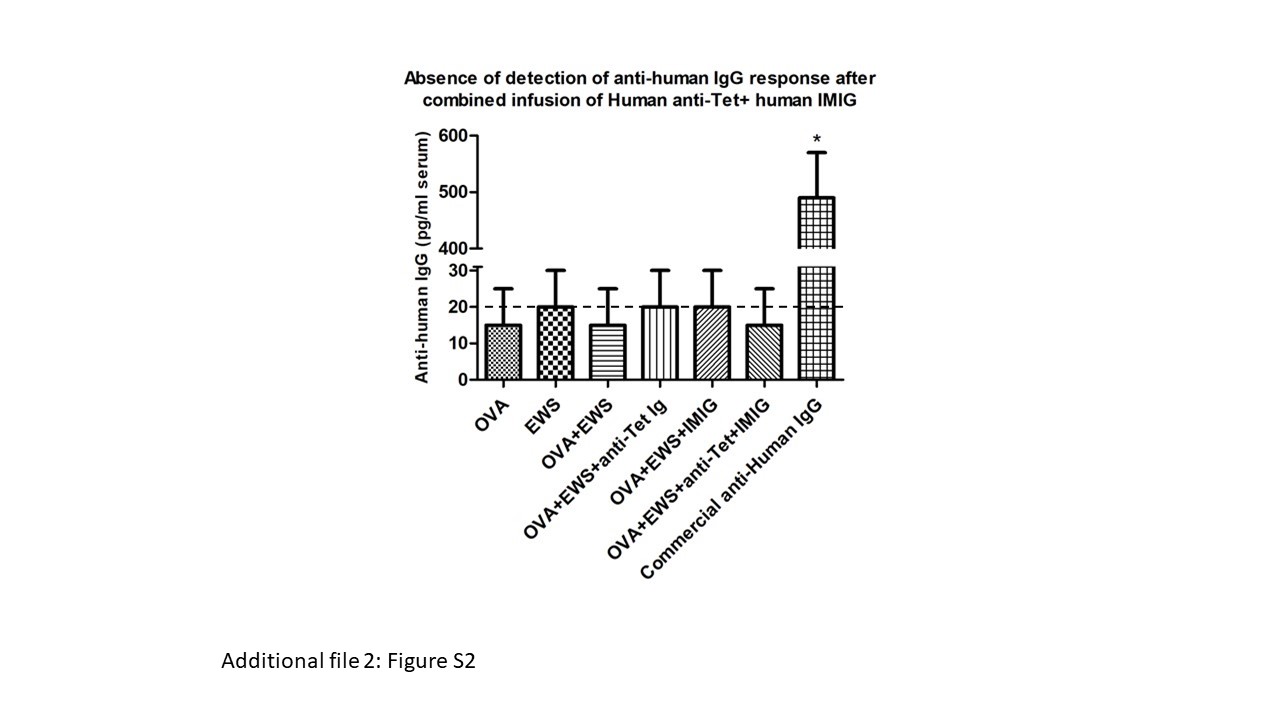

Supplement: Supplementary file 2 — Additional file 2: Figure S2. Absence of detectable mouse anti-human IgG responses in mice receiving heterologous (human) Anti-Tet immune Ig, IMIG, or a mixture of the two (at separate sites). 100 μl serum (diluted 1:3) was assayed in duplicate from each of the mice at sacrifice (after 5 injections) shown in Figure 3, with ELISA plates coated with human IgG (100 ng/well), and commercial goat anti-mouse Ig-HRP as developing agent (1:1000). A commercial mouse anti-Human IgG was used as a positive control (ThermoFisher, 1:1000). Data show group means ± SD. The dotted line shows the detection limit in the assay (20 pg/ml). [file 13223_2019_393_MOESM2_ESM.jpg]

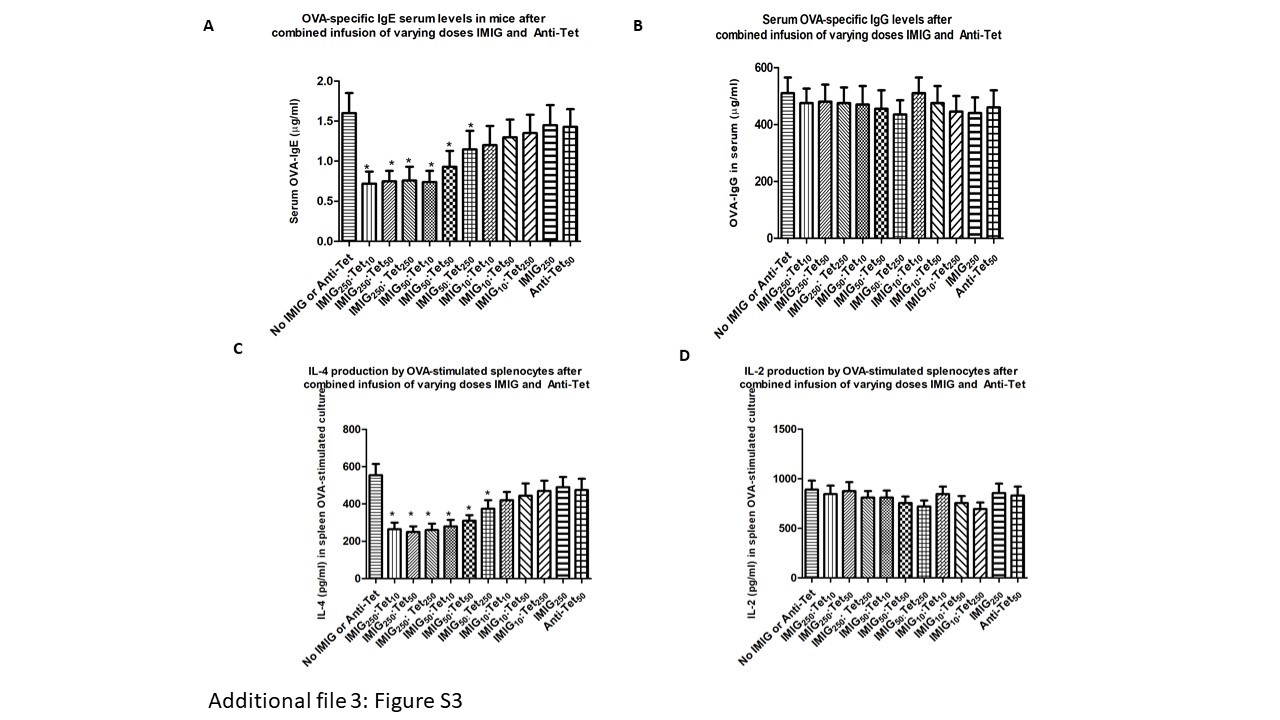

Supplement: Supplementary file 3 — Additional file 3: Figure S3. Comparison of attenuation of OVA-specific immune response (compare with Fig. 3) in mice receiving different doses, ranging from 250 μg/mouse to 10 μg/mouse, of human IMIG or anti-Tet immune Ig given im at weekly intervals. Control groups received the highest dose of IMIG (250 μg/mouse) or an intermediate dose of anti-Tet Ig (50 μg/mouse) alone. Data show mean ± SD of Ig levels in serum, or cytokines at 72 h in culture supernatants. In subsequent studies we have routinely used IMIG (75 μg/mouse) and anti-Tet Ig (10 μg/mouse). *, p < 0.05 compared with mice receiving no Human IMIG or anti-Tet Ig. [file 13223_2019_393_MOESM3_ESM.jpg]

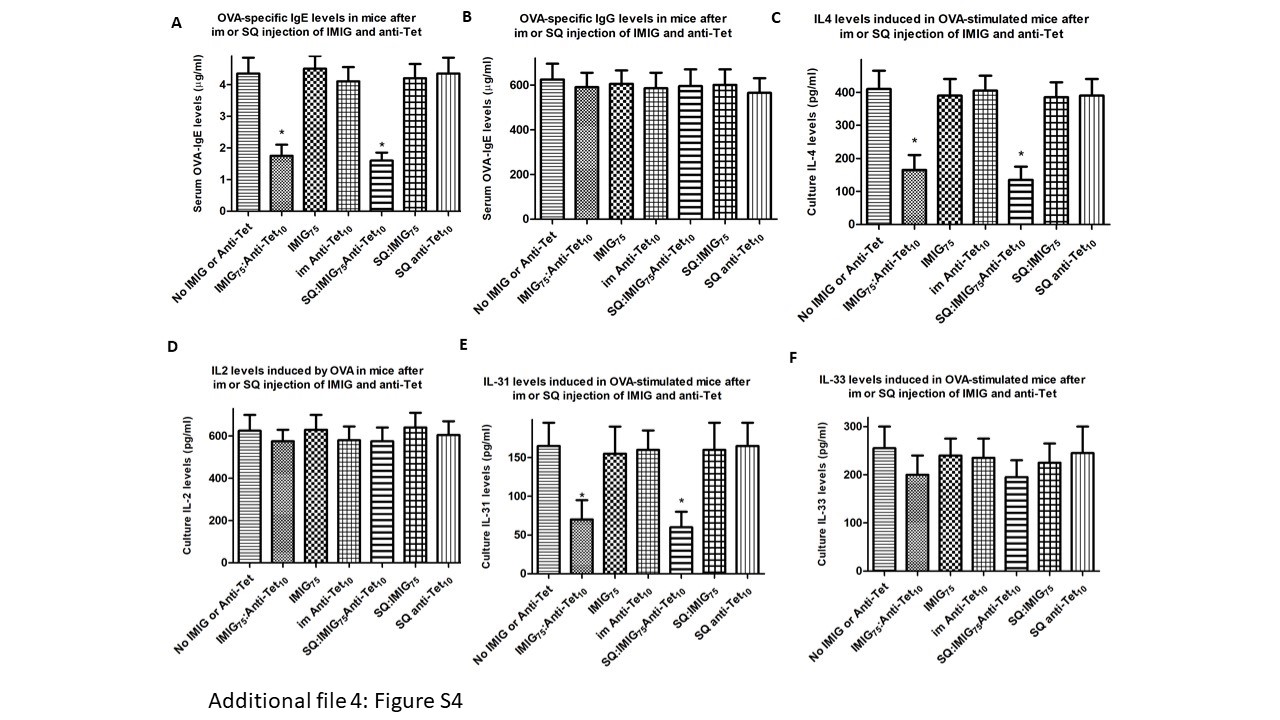

Supplement: Supplementary file 4 — Additional file 4: Figure S4. Comparison of attenuation of OVA-specific immune response in mice (see Fig. 3) receiving combination treatment with human IMIG or anti-Tet immune Ig each given via either an intramuscular or subcutaneous route of administration at weekly intervals. Control groups received either the IMIG or anti-Tet alone, again via either of these two routes. Data show mean ± SD of Ig levels in serum, or cytokines at 72 h in culture supernatants. Note that cultures in this instance were also assayed for IL-31 and IL-33, given the recent interest in their use as markers of allergic inflammation. *, p < 0.05 compared with mice receiving no Human IMIG or anti-Tet Ig. [file 13223_2019_393_MOESM4_ESM.jpg]
